# Supplementary material for: An Engineered Nanocomplex with Photodynamic and Photothermal Synergistic Properties for Cancer Treatment
Source: Int J Mol Sci. 2022 Feb 18;23(4):2286. doi: 10.3390/ijms23042286 (PMC8874418; doi:10.3390/ijms23042286)
Supplement: Supplementary file 1 [file ijms-23-02286-s001.zip › ijms-1600447-supplementary.pdf]

International Journal of
Molecular Sciences

MDPI

Supplementary

An Engineered Nanocomplex with Photodynamic and Photothermal Synergistic Properties for Cancer Treatment

Eli Varon,[1,2] Gaddi Blumrosen,[1,2] Moshe Sinvani,[1,2] Elina Haimov, [1,2] Shlomi Polani, [2,3] Michal Natan,[1,2] Irit Shoval,[4] Avi Jacob,[4] Ayelet Atkins,[2] David Zitoun,[2,3] and *Orit Shefi [1,2,5]

[1] Faculty of Engineering, Bar-Ilan University, Ramat Gan 5290002, Israel.
[2] Bar-Ilan Institute of Nanotechnology and Advanced Materials, Bar-Ilan University, Ramat Gan 5290002, Israel.
[3] Department of Chemistry, Bar-Ilan University, Ramat Gan 5290002, Israel.
[4] Faculty of Life Sciences, Bar-Ilan University, Ramat Gan 5290002, Israel.
[5] Gonda Multidisciplinary Brain Research Center, Bar-Ilan University, Ramat Gan 5290002, Israel.

* Correspondence: orit.shefi@biu.ac.il.

Citation: Varon, E.; Blumrosen, G.; Sinvani, M.; Haimov, E.; Polani, S.; Natan, M.; Shoval, I.; Jacob, A.; Atkins, A.; Zitoun, D.; et al. An Engineered Nanocomplex with Photodynamic and Photothermal Synergistic Properties for Cancer Treatment. *Int. J. Mol. Sci.* **2022**, *23*, 2286. https://doi.org/10.3390/ijms23042286

Academic Editor: Antonio Di Stefano

Received: 31 January 2022
Accepted: 17 February 2022
Published: 18 February 2022

Publisher's Note: MDPI stays neutral with regard to jurisdictional claims in published maps and institutional affiliations.

Copyright: © 2022 by the authors. Licensee MDPI, Basel, Switzerland. This article is an open access article distributed under the terms and conditions of the Creative Commons Attribution (CC BY) license (https://creativecommons.org/licenses/by/4.0/).

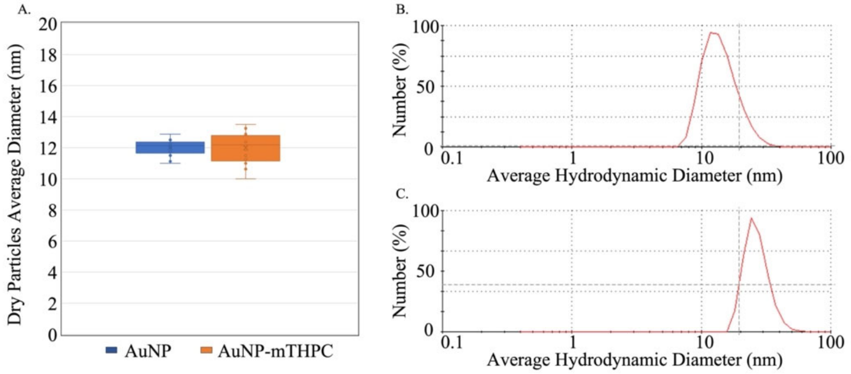

Figure S1 | Dry and hydrodynamic sizes of AuNP and AuNP-mTHPC. (A) The average dry size of AuNP and AuNP-mTHPC acquired through TEM, (n=20). Hydrodynamic size distribution of nanoparticles was measured using DLS. The diameter of AuNP (B) was 52 ± 4 nm, while that of mTHPC-coated AuNP (C) was 77 ± 8 nm.

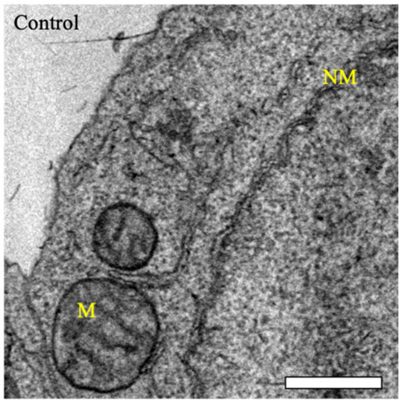

Figure S2 | SH-SY5Y untreated TEM images (A) Electron micrograph of SH-SY5Y untreated cells. The image shows mitochondria (M) that do not contain AuNP-mTHPC. The plasma membrane and the nuclear membrane (NM) are well shaped. Scale bar = 0.5 μm.

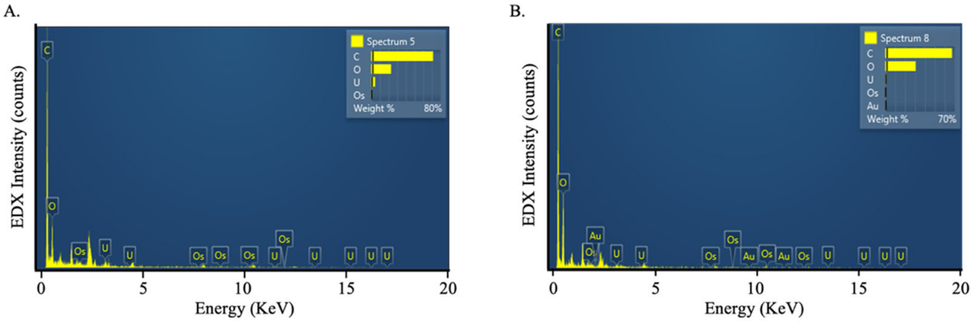

Figure S3 | EDX analysis of SH-SY5Y cells. Energy disperse spectroscopic spectra (A) Untreated cells (B) Cells treated with 1.2 μM AuNP-mTHPC. The element weight percent of C, O, Os, Au, and U is displayed.

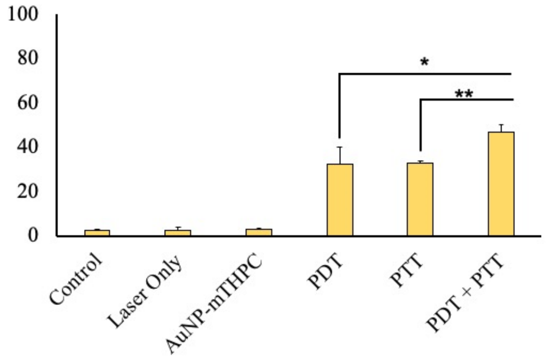

Figure S4 | Cell death induced by laser irradiation after 24 h incubation with AuNP−mTHPC complex. (A) Cell viability of SH-SY5Y cells with 1.2 μM AuNP-mTHPC and illuminated under 650 nm laser (PDT) at 6 mW/cm² for 4 min or illuminated under 532 nm laser (PTT) at 15 mW/cm² for 4 min. The combination of PDT PTT was conducted using 2 min of each laser. Average quantification of three experiments is presented in the plots (mean ± STDEV). There were significant differences in the relative levels control and PDT and PTT in the experiments. **P < 0.01, *P < 0.05.

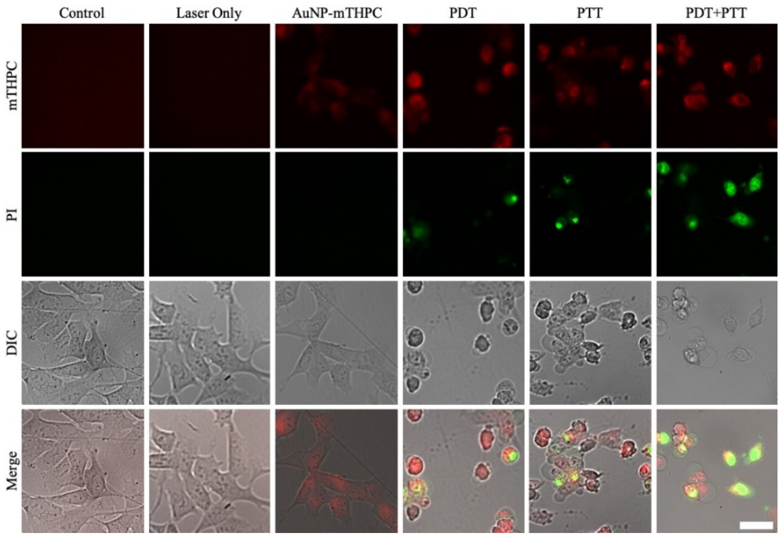

Figure S5 | Microscopy Images of SH-SY5Y cells after PDT/PTT treatment (A) Confocal microscopy of 1.2 μM AuNP-mTHPC (red) and PI (green) labeled SH-SY5Y cells after laser irradiation under 650 nm laser (PDT) at 6 mW/cm² for 4 min or/and illuminated under 532 nm laser (PTT) at 15 mW/cm² for 4 min. Scale bar = 50 μm.
